# Editorial: The role of stress proteins in plants under abiotic stress

**DOI:** 10.3389/fpls.2023.1193542

**Published:** 2023-05-04

**Authors:** Peng Zhou, Steffen P. Graether, Longxing Hu, Wanjun Zhang

**Affiliations:** ^1^ School of Agriculture and Biology, Shanghai Jiao Tong University, Shanghai, China; ^2^ Department of Molecular and Cellular Biology, University of Guelph Guelph, Guelph, ON, Canada; ^3^ Department of Grassland Science, College of Agronomy, Hunan Agricultural University, Changsha, China; ^4^ College of Grassland Science and Technology, China Agricultural University, Beijing, China

**Keywords:** stress proteins, functional analysis, post-translational modification, bioinformatics analysis, the stress-associated protein, the stress-activated protein kinase, late embryogenesis abundant protein

Extreme environmental conditions pose significant challenges to plant survival and productivity. Over time, plants have evolved a myriad of defense mechanisms to mitigate the detrimental effects of adverse conditions. These mechanisms induce the production of a plethora of protective proteins, which play a crucial role in preserving the normal physiological and biochemical processes of plants under stress. In-depth investigation of these stress proteins may uncover significant insights into their potential applications in agricultural production.

Various stress proteins have been identified, such as late embryogenesis abundant proteins (LEA proteins, including dehydrins), reactive oxygen species (ROS) scavenging enzymes, heat shock proteins (HSPs) that serve as molecular chaperones to preserve enzyme activity and protein structure, and enzymes responsible for maintaining plant redox status and eliminating oxygen free radicals. Current research has delved into the significance and mechanisms of these stress proteins, expanding our understanding of both their depth and scope. This knowledge has been extensively applied to the study of numerous plant species, ultimately contributing to the enhancement of agricultural production and improvement of ecological environments in the long term.

This Research Topic has been meticulously organized, featuring three original research articles and one comprehensive review, designed to emphasize recent advancements in the following areas: (1) in-depth functional analysis of stress proteins, (2) regulatory networks of stress proteins, (3) bioinformatics analysis of stress proteins within the context of big data, and the cross-species evolutionary relationships of stress proteins.

## In-depth functional analysis of stress proteins

A wide array of stress proteins has been identified in various plant species. However, the precise mechanisms through which some of these proteins function within plant cells remain elusive. One such example is the stress-associated protein (SAP) family. SAP family genes have been isolated from diverse plants, including *Arabidopsis thaliana*, *Populus euphratica*, and *Populus trichocarpa*, yet their mode of action within plant cells has not been reported. What is known is that SAPs represent a novel class of highly conserved zinc-finger proteins across different plant species and are intimately associated with stress tolerance in plants.

Park et al. employed RNA interference technology to knock down an SAP gene, designated as *PagSAP11*, in hybrid poplar (*Populus alba × Populus tremula* var. *glandulosa*). Their findings revealed that the knockdown of *PagSAP11* augmented poplar tolerance to drought stress and promoted the branching of lateral shoots. Additionally, the branching phenotype was associated with the upregulated expression of several genes that are implicated in axillary bud outgrowth and cell proliferation. This study holds considerable value in further elucidating the mechanisms of SAPs within plant cells.

## Regulatory networks of stress proteins

At the molecular level, plant response mechanisms to stress are multifaceted. Plants can react to stress by activating transcription factors, which in turn regulate the up or downregulation of specific genes. Alternatively, a single gene can produce various mature functional proteins to adapt to environmental changes. Post-translational modification (PTM) represents a crucial regulatory mechanism that enables plants to respond to stress.

Xiao et al. conducted a comprehensive review of recent findings regarding the interplay between histone (de)methylation and osmotic stress. They also underscored the influence of stress on histone methylation profiles and the role of histone methylation in optimizing plant performance under stress conditions. Their work offers valuable insights into how histone modifications regulate plant responses to stress.

## Bioinformatics analysis of stress proteins

Advancements in computer technology and the availability of various species’ genome databases have facilitated in-depth investigations of stress protein families. Genome-wide analyses of genes encoding stress proteins in specific species can provide novel insights into the distribution of gene families within genomes and the evolutionary relationships of these gene families, even between different species. In conjunction with RNA-seq data, researchers can identify more potent genes within the same gene family.

The stress-activated protein kinase (SAPK), also known as SnRK2, responds to abiotic stress through abscisic acid (ABA)-dependent or independent signaling pathways. SAPKs are promising candidate genes for enhancing plant stress resistance. Xing et al. identified 10 *LpSAPKs* in perennial ryegrass (*Lolium perenne* L.), with most *LpSAPKs* demonstrating responsiveness to various abiotic stressors. Employing a yeast ectopic expression system, the authors further investigated the role of *LpSAPK9* in plant drought tolerance.

LEA proteins are highly expressed in plants under diverse abiotic stresses, bolstering plant resistance. The LEA protein family encompasses several subfamilies, including LEA_2, which is distinct from other LEA proteins and plays critical roles in plant stress tolerance. To date, the function of LEA_2 remains inadequately understood. Zhang et al. identified 155 members of *LEA_2* from the alfalfa (*Medicago sativa* L.) genome. Bioinformatics analysis revealed that *MsLEA_2* genes are distributed across all 32 chromosomes, with 85 genes present in gene clusters, accounting for 54.83%. Cis-element analysis confirmed that the promoter region of *MsLEA_2* is rich in ABRE, MBS, LTR, and MeJARE. Additionally, in combination with RNA-seq data, the authors found that *MsLEA_2* genes exhibit stress resistance potential under abiotic stress. These findings hold promise for future functional analyses of LEA_2 proteins.

## Author contributions

PZ wrote the paper. SG, LH, and WZ had revised it. All authors contributed to the article and approved the submitted version.

